# Structural Insights into the Mechanism of Phosphoregulation of the Retinoblastoma Protein

**DOI:** 10.1371/journal.pone.0058463

**Published:** 2013-03-14

**Authors:** Ekaterina P. Lamber, Fabienne Beuron, Edward P. Morris, Dmitri I. Svergun, Sibylle Mittnacht

**Affiliations:** 1 Division of Cancer Biology, Institute of Cancer Research, London, United Kingdom; 2 Division of Structural Biology, Institute of Cancer Research, London, United Kingdom; 3 European Molecular Biology Laboratory, Hamburg Outstation, Hamburg, Germany; 4 Research Department of Cancer Biology, UCL Cancer Institute and National Institute for Health Research, University College London Hospitals Biomedical Research Centre, University College London, London, United Kingdom; University of Queensland, Australia

## Abstract

The retinoblastoma susceptibility protein RB1 is a key regulator of cell proliferation and fate. RB1 operates through nucleating the formation of multi-component protein complexes involved in the regulation of gene transcription, chromatin structure and protein stability. Phosphorylation of RB1 by cyclin-dependent kinases leads to conformational alterations and inactivates the capability of RB1 to bind partner protein. Using small angle X-ray scattering in combination with single particle analysis of transmission electron microscope images of negative-stained material we present the first three-dimensional reconstruction of non-phosphorylated RB1 revealing an extended architecture and deduce the domain arrangement within the molecule. Phosphorylation results in an overt alteration of the molecular shape and dimensions, consistent with the transition to a compact globular architecture. The work presented provides what is to our knowledge the first description of the relative domain arrangement in active RB1 and predicts the molecular movement that leads to RB1 inactivation following protein phosphorylation.

## Introduction

The retinoblastoma tumour susceptibility protein (RB1) plays an important role in regulating cell cycle progression, cell survival and differentiation [1], [2]. Heritable mutations in the RB1 encoding gene greatly increase the risk for development of the paediatric eye tumour retinoblastoma and significantly enhance the overall lifetime risk for the development of other cancers [3], [4], [5]. RB1 is mutated or lost in other common cancers, including small cell lung cancer and breast, and inactivated through binding and destabilization by the human papillomavirus (HPV) transforming protein E7 in the majority of cervical cancers [6]. RB1 function is thought to be compromised by mutation of the upstream regulatory network in the majority of sporadic cancers [7], [8].

RB1 operates through interaction with cellular proteins. More than 110 different proteins have been shown to interact with RB1 [9], including several DNA binding transcription factors [9], [10], as well as proteins with multiple functions in chromatin modification [2], [9], and components of the ubiquitin ligase machinery [11]. RB1 in cells is found in multi-component protein assemblies and *in vitro* is capable of supporting protein interactions through a minimum of four independent surfaces suggestive of its functioning as a scaffold involved in nucleating complex formation [2]. Phosphorylation of RB1 by members of the proline-directed family of cyclin-dependent Serine (Ser) Threonine (Thr) protein kinases inactivates the ability of RB1 to interact with partner proteins [7], [12], presumably instigating fragmentation of the RB1-containing protein assemblies.

RB1 belongs to a family of proteins including the RB1 paralogues RB1L1/p107 and RB1L2/p130 that share overall sequence conservation, including substantial sequence identity within a centrally located pocket domain [13]. Through their central pocket domain RB family proteins support the interaction with proteins containing a LeuXCysXGlu (LXCXE) short linear motif, found in viral transforming proteins including the HPV E7 protein but also cellular proteins [14], and the interaction with proteins containing a GluXXXAspLeuPhe (EXXXDLF) motif, found in the C-terminal transactivation region of E2 family transcription factors (E2Fs) [15], [16]. RB1 contains two further regions known for their involvement in protein interactions, the N-terminal domain, RB-N, which is related in architecture to RB-P and features a protein interaction surface analogous to that involved in LXCXE binding in the pocket [17], and the C-terminal domain, RB-C, involved in associating with the dimer surface resulting form association of the E2Fs with their partner dimer proteins (DPs) [18].

**Figure 1:**
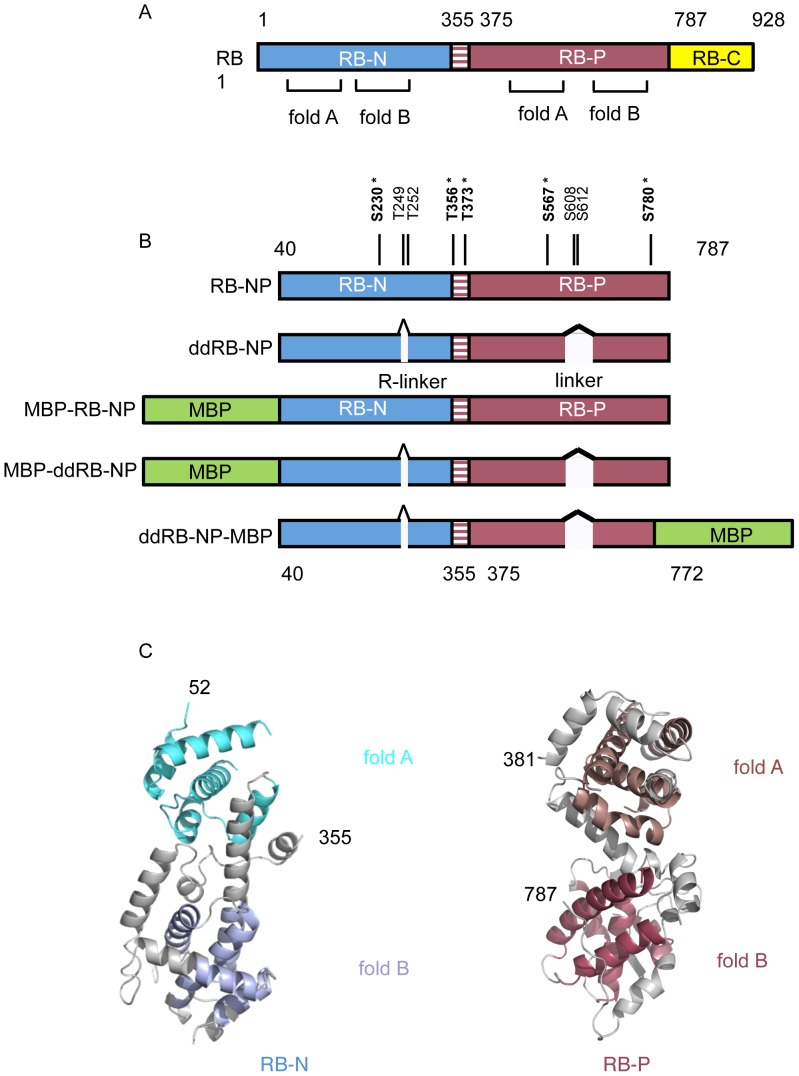
RB1 architecture and study design. **A.** Schematic of RB1 domain structure. RB1 NH2-terminal domain (RB-N, light blue), RB1 pocket-domain (RB-P, raspberry), the position of the twin cyclin folds which form the core of each domain is indicated, RB1 C-terminal region (RB-C, yellow)**. B.** RB1 constructs used in this study indicating the range of amino-acids covered. In the MBP-RB-NP and MBP-ddRB-NP constructs maltose binding protein (MBP, green) is coupled to the N-terminus of the RB construct, while in ddRB-NP-MBP it is coupled to the C-terminus. In the ddRB-NP, MBP-ddRB-NP and ddRB-NP-MBP constructs two interstitial regions were deleted, corresponding to residues 250–269, the arginine-rich linker (R-linker) of the RB-N domain, and residues 579–643, corresponding to the pocket linker connecting RB-P domain pocket lobes (P-linker). The positions of cyclin-dependent kinase consensus sites in RB-NP are indicated, with sites retained in the ddRB-NP, MBP-ddRB-NP and ddRB-NP-MBP constructs bold and starred. **C.** Atomic models of the RB-N and RB-P domains, shown in ribbon representations. RB-N left, RB-P right. Cyclin-fold helixes are coloured, RB-N A-fold in cyan, RB-N B-fold in light blue, RB-P A-fold in dark salmon, RB-P B-fold in pink, other helixes and visible loops are shown as grey.

Although atomic resolution structures of the various RB1 functional domains have been determined (Figure 1C), how these domains and their respective protein interaction surfaces are arranged in the active molecule is not known. In the work presented here we characterize an RB1 entity containing the RB-N and RB-P domains using small angle X-ray scattering (SAXS) combined with single particle analysis of transmission electron microscope (TEM) images of negatively stained material. The work allows the deduction of the domain arrangement in the active unphosphorylated form and permits prediction of the cause and mechanics of the conformational response leading to functional inactivation by cyclin-dependent kinase phosphorylation.

## Results

### Characterization of RB1 Multi-domain Assemblies by Small Angle X-ray Scattering

To characterize the domain arrangement within RB1 we generated a series of derivatives of the human protein (Figure 1A) which are illustrated in Figure 1B. The first (RB-NP) is made up from the structured RB-N and RB-P domains connected by the 18 amino acid linker (residues 356–374) that joins these two domains. This was coupled through its N-terminus to hexahistidine-tagged maltose binding protein (MBP) using a tobacco etch virus (TEV) protease-cleavable linker. We excluded the RB1-C region of RB1 from this and other constructs since the RB1-C region was previously shown to be unfolded in an empty/unliganded state [18]. We also generated additional constructs in which two interstitial regions (residues 250–269 in RB-N and residues 579–643 in RB-P, which both are absent in the atomic resolution structures of these respective domains) had been removed (ddRB-NP). Finally, we generated a version of ddRB-NP in which the MBP-tag linked to a TEV protease sequence was fused to residue 772 of ddRB-NP (ddRB-NP-MBP). For structural analysis we made use of the products in which the fused MBP-tags were removed by TEV protease treatment (RB-NP and ddRB-NP) as well as the products in which they were retained (MBP-RB-NP, MBP-ddRB-NP and ddRB-NP-MBP).

Multi-angle light scattering (MALS) measurements (Table S1 and Figure S1A) gave estimated molecular masses for each of these preparations which were consistent with their respective predicted molecular masses, indicating that they are predominantly monomeric. Furthermore, cross-linking (Figure S1B) performed using ddRB-NP at high concentrations, as used in SAXS data collection, revealed only minor tendencies for dimer formation. Accordingly, structural measurements made in solution can be taken to represent the monomeric state for each of these proteins.

**Figure 2:**
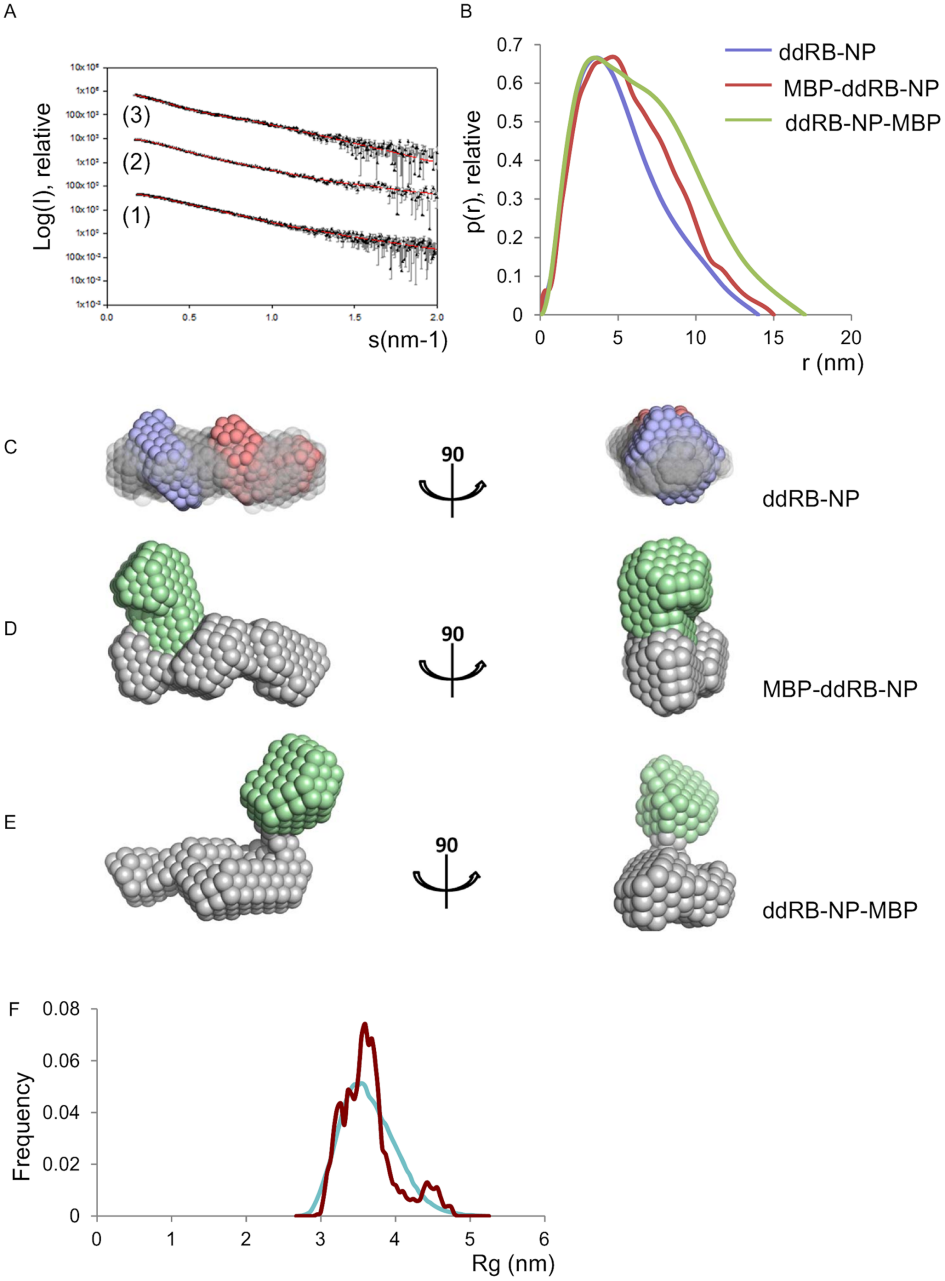
Characterisation of RB1 derivatives by small-angle X-ray scattering. **A**. Experimental and calculated scattering patterns of ddRB**-**NP (1), MBP-ddRB**-**NP (2), ddRB**-**NP-MBP (3). Experimental SAXS data as black dots with black error bars. Lines (red) represent the fits from *ab initio* models shown in **C** (ddRB-NP), **D** (MBP-ddRB-NP) and **E** (ddRB-NP-MBP). The logarithm of the scattering intensity is plotted as a function of momentum transfer, s = 4πsin(θ/2)/λ where θ is the scattering angle and λ is the wavelength of the X-rays (1.5 Å). **B.** Distance distribution functions for ddRB-NP, MBP-ddRB-NP and ddRB-NP-MBP. **C.** Averaged *ab initio* models for ddRB-NP obtained using DAMMIN (grey semi-transparent spheres) and MONSA (RB-N blue spheres, RB-P red spheres) superimposed. The models are shown in two different views rotated by 90°. **D., E.** *Ab initio* models of MBP-ddRB-NP (**D**) and ddRB-NP-MBP (**E**) obtained by MONSA. MBP is shown as green, ddRB-NP as grey spheres. The models are viewed as in **C**. **F.** Radius of gyration (*R_g_*) distribution obtained by EOM for ddRB-NP. Distributions correspond to a random pool of 10.000 generated structures (blue) and the EOM optimized ensemble (red).

The small angle scattering pattern and the distance distribution function P(r) for ddRB-NP and its MBP-tagged derivatives are presented in Figure 2A and B, with derived parameters presented in supplementary Table S2. Plots of the Guinier-region for each construct are shown in Figure S1C confirming linear data distribution indicative that the sample is monodisperse. The overtly skewed shape of the calculated distance distribution (Figure 2B) indicates that all the preparations have elongated as opposed to globular shape, including the untagged ddRB-NP. Furthermore, these data indicate a considerable increase in elongation for ddRB-NP-MBP (Dmax 17±1 to 18±1 nm) over ddRB-NP (Dmax 14±1 nm) (see Table S2), consistent with the MBP-tag forming a C-terminal extension in this protein construct. Only a modest increase in Dmax was observed for MBP-RB-NP over ddRB-NP, indicating a potentially partly lateral positioning of the N-terminal MBP-tag.

Use of the experimentally determined SAXS measurements to compute a low resolution *ab initio* model in DAMMIN [19], (Figure 2C), reveals an elongated shape for ddRB-NP, consistent with an extended non-globular architecture of this domain assembly. To compute the putative positions of the RB1 domains within the experimentally determined shape, we used MONSA [19] an extended version of DAMMIN capable for the multiphase modelling. A tentative *ab initio* model in which the domains are arranged sequentially is shown on Figure 2C: this model fitted the experimental SAXS data with χ = 0.8.

We also performed MONSA-based *ab initio* modelling for the MBP-tagged preparations, yielding probabilistic models in line with a lateral as opposed to a terminal location of MBP in MBP-ddRb-NP (Figure 2D). Conversely, a tentative *ab initio* model calculated by MONSA for ddRB-NP-MBP depicts MBP to a terminally protruding position (Figure 2E).

To assess the impact of the linker deletions within RB-N and RB-P (residues 250–269 in RB-N and 579–643 in RB-P) on the structure of the RB protein, we performed SAXS using the derivative preparation in which these regions were left in place (RB-NP and MBP-RB-NP). MALS results for these preparations are shown in Table S1 and Guinier region plot in Figure S1C, indicating that these preparations also exist predominantly as monomers in solution with essentially monodisperse distribution in the samples subjected to SAXS measurements. Importantly, comparison of scattering patterns or distance distribution functions for RB-NP and MBP-RB-NP with scattering patterns or distance distribution for ddRB-NP and MBP-ddRB-NP (Figure S2) did not reveal any significant difference, with data distributions being the same within experimental error. Hence deletion of these linkers does not affect the shape of RB-NP as measured by SAXS and therefore the conclusions and modelling derived from the linker-deleted variants is most likely relevant to the full-length protein assemblies.

To probe for inherent flexibility within ddRB-NP, which could interfere with *ab initio* shape determination, we employed the ensemble optimization method (EOM) which quantitatively characterizes the conformational space of proteins in solution from SAXS data [20]. EOM analysis revealed an Rg distribution of the reconstructed ensemble for ddRB-NP that is essentially narrower than the Rg distribution of the random pools, indicating that the protein possesses limited flexibility, confined to an Rg differential of 10 Å or less (Figure 2F). Docking the atomic structures for RB-N and RB-P into the *ab initio* model of ddRB-NP (Figure 2C) yields a rigid body model, which provided a fit to the experimental data with discrepancy χ  = 1.02. In recently published work [21] substantial conformational heterogeneity was observed with an RB1 fragment preparations similar to ddRB-NP suggesting a mixture of “closed” and “opened” forms. To address directly the possibility of a mixture of “closed” and “opened” forms, the experimental data from ddRB-NP were fitted by a linear combination of the rigid body model and of the “closed” conformation reported in [21]. This analysis, carried out using OLIGOMER [22], did not improve the fit, the experimental data yielding the volume fraction of the closed conformation being equal to zero. Taken together, the EOM analysis and the good fit between a single rigid body model and the experimental scattering data, suggest a preferred and stabile extended "opened" conformation of the RB-N and RB-P domains in ddRB-NP. We note, however, presence of a small peak at higher Rg, which could indicate some minor presence of species with significantly larger dimensions.

### Refinement of the Domain Arrangement of RB1 by Single Particle Analysis of Electron Microscope Images

**Figure 3:**
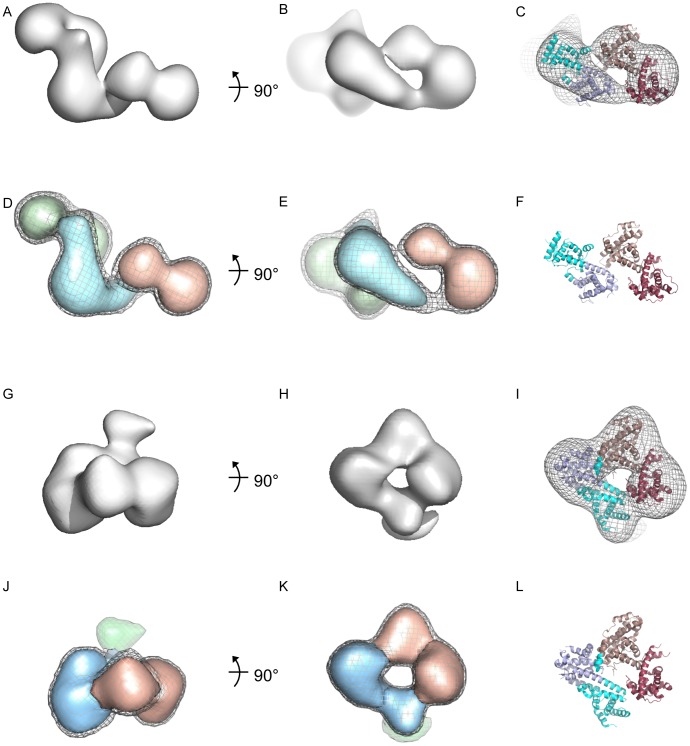
Single particle analysis of electron microscope images of MBP-ddRB-NP. A.-F. 3D reconstruction of unmodified MBP-ddRB-NP. **A.**, **B**. Single particle reconstruction for unmodified MBP-ddRB-NP. Calculated density map of MBP-ddRB-NP, shown as surface representations in grey related by a 90^o^ rotation. **C.** 3D reconstruction in mesh representation oriented as in **B** with the docked structures of the RB-N and RB-P domains (PDB codes 2QDJ and 3POM) shown as cartoons colour-coded as follows: RB-N domain lobe A -cyan, lobe B -light blue; RB-P domain lobe A -dark salmon and lobe B – pink. **D., E.** Segmented densities shown as solid surface representation with overlaid surface representation of the unmodified RB-NP 3D reconstruction in mesh. The density attributed to the MBP tag is shown in light green, that attributed to RB-N in light blue and to RB-P in light pink. **F.** Docked structures of the RB-N and RB-P domains (PDB codes 2QDJ and 3POM) without density mesh, shown as cartoons and colour-coded as in C. **G.–L.** 3D reconstruction of phosphorylated MBP-ddRB-NP. **G., H**. 3D reconstruction shown as a grey surface in two orthogonal views. **I.** 3D reconstruction in mesh representation oriented as in **H** with the docked structures of inactive RB-NP (PDB code 4ELJ) shown as cartoons colour-coded as follows:-. RB-N domain lobe A -cyan, lobe B -light blue; RB-P domain lobe A -dark salmon and lobe B – pink. **J., K.** Segmented densities shown as solid surface representation with overlaid surface representation of the 3D reconstruction in mesh**.** Same colour coding as in **D** and **E. L.** Docked structures of inactive RB-NP (PDB code 4ELJ) without density mesh, shown as cartoons colour-coded as in I.

**Figure 4:**
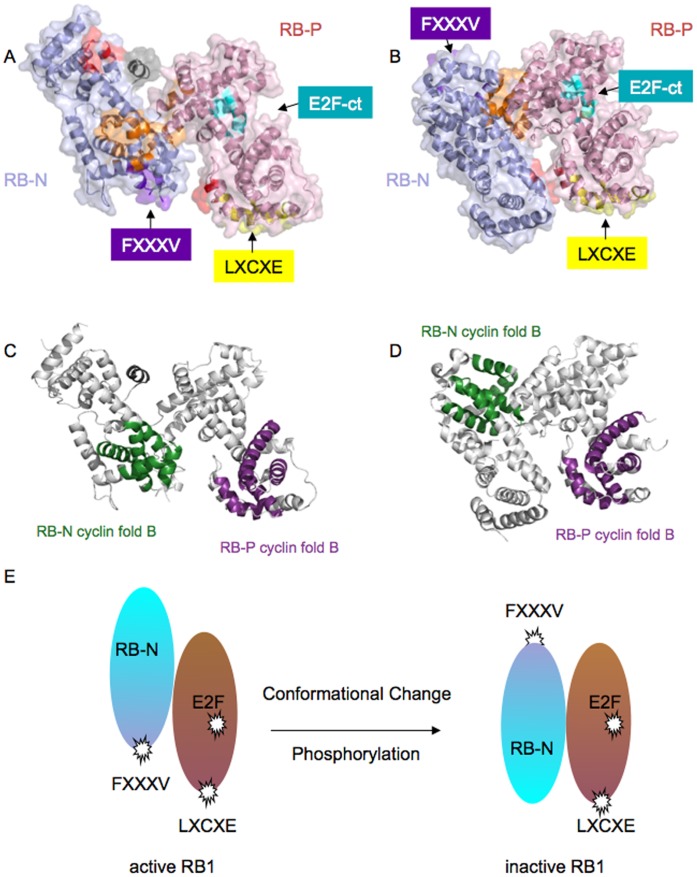
Binding surfaces positioning in the active and inactive structure, and predicted molecular movement to yield inactive RB1. A., B. Relative orientation of the functional surfaces in the model of active, nonphosphorylated (A) and inactive, phosphorylated (B) RB1. Cartoon representation of Rb-NP with overlaid transparent surface with RB-N in light blue, RB-P in light-pink. The residues involved in docking LXCXE are shown in yellow, those forming the FXXXV motif are shown in purple and those for EXXXDLFD in cyan. The residues 346–355 which form a helix in unmodified RB-N but are disordered in inactive RB-NP are represented in dark grey [17], [21], amino acid groups involved in RB-N:P interphase interaction in the inactive conformation in red ([RB-N K136, D139, T140, T142, D145], [RB-P Q736, E737, K740, K729]) and orange [(RB-N L161, K164, L206-E209, L211-I213, F216, E282, E287, N290, N295] [RB-P Q736, E737, K740, K729]). **C., D.** Cartoon representation of active, nonphosphorylated, (C) and inactive, phosphorylated (D) RB1. RB-N B-fold is coloured in green and RB-P B-fold in purple (this different colour scheme has not been used elsewhere in the paper and is only used here for clarity). The residues 346–355 which are structured in unmodified RB-N and unstructured in inactive RB-NP are represented in dark grey. **E.** Predicted molecular movement yielding conformational RB1 inactivation. Note surfaces involved in binding LXCXE motif proteins in RB-P (salmon/pink) and the homologous surface involved in FXXXV binding in RB-N (cyan/blue) are collinear in the active (left) but not inactive form (right).

While the analysis and modelling of the SAXS data shows that RB-NP adopts an elongated architecture, it provides only approximate and tentative information on the relative orientations of the RB and NP domains. To obtain a higher resolution description of RB and more exact information on the domain arrangement we performed electron microscopy and single particle analysis on negatively stained material. For this purpose we initially focused on the MBP-ddRB-NP protein as its increased molecular mass compared to derivatives without MBP-tag makes it more suitable for imaging by TEM and subsequent analysis. Representative molecular images and *ab intio* class averages derived from MBP-ddRB-NP are shown in Figure S3A and B. The majority of such images are substantially elongated, consistent with the molecular shape identified in the SAXS analysis (Figure 2D) projected normal to its long axis. The remaining less markedly elongated molecular images are consistent with projections approximately in the direction of the long axis. Three-dimensional analysis from a dataset of 5829 molecular images was performed using the SAXS envelope for MBP-ddRB-NP low pass filtered to 40 Å as an initial reference (Figure S4A(i)). The analysis, documented in Figure S3C–E, resulted in a three-dimensional reconstruction (Figure 3A, B) with an estimated resolution of ∼27 Å which replicates the elongated appearance of the SAXS envelope (Figure 2D) but is characterised by a substantially increased level of detail. This level of detail in the EM map allowed it to be confidently segmented into three components using the Chimera segmentationwhich served as a basis for domain assignment and rigid body docking of the RB and MBP domains. A preferred fit optimised for correlation of the available atomic resolution structures with the experimental model space, with minimised distances between domain termini and their adjoining residues, is shown in Figure 3C, F. This model features a recessed, parallel (A-B:A-B) arrangement of RB-N and RB-P in which their respective B-lobe cyclin wedges, known for involvement in short motif interaction, lie within close vicinity (Figure 4C). *Ab initio* protein structure prediction using Protein Homology/analogy Recognition (PHYRE) vs2.0 [23] independently identified a highly congruent positioning for RB-N and RB-P suggesting a A-B:A-B lobe orientation as the most likely tertiary structural arrangement based on sequence (Figure S5). An alternative rigid body fit to the EM density map is formally possible whereby RB-N and RB-P are arranged in an anti-parallel (B-A:A-B) orientation (Figure S6A). However this model was judged to be significantly less satisfactory because the adjoining residue pairs of the individual domains are separated by a considerable distance (Figure S6A).

### Effect of Phosphorylation on Molecular Shape and Envelope

RB1 is inactivated by phosphate modification on proline-directed sites, with phosphorylation disabling the ability of RB1 to sustain interaction with partner proteins [24], [25], [26], [27], [28], reviewed in [29]. Recently, an atomic resolution structure has been obtained for the presumed inactive form of ddRB-NP carrying phosphate modification on two residues (Thr356 and Thr373) within the segment (residues 356–376) that joins RB-N to RB-P [21]. In this study the arrangement of domains in phosphorylated ddRB-NP results in a significantly less elongated structure compared with the one we have deduced for corresponding unphosphorylated ddRB-NP (Figure 3F). To further investigate the effects of phosphorylation on the conformation in solution of ddRB-NP we treated preparations of MBP-ddRB-NP with KSHV encoded D-cyclin (Kcyclin)/CDK6 complex. Tandem mass spectrometry (MS/MS) of the phosphorylated material indicated ready modification of both Thr356 and Thr373 (Figure S7B, C). Two other proline-directed sites (Ser230 and Ser780) present in ddRB-NP were not modified, although peptides containing these residues in their unmodified form were readily identified in the MS/MS profile. Absence of phosphorylation on these sites most likely is explained by the principle structural inaccessibility of S230 [17] and a requirement for additional substrate recognitions sequences in RB-C for modification of Ser780 by cyclin/CDKs [30]. MALS-based molecular size assessment indicates that like unmodified MBP-ddRB-NP phosphate-modified MBP-ddRB-NP also preferentially exists in a monomeric state in solution (Figure S1B).

Analysis of Kcyclin/CDK6-modified MBP-ddRB-NP preparations by TEM revealed a visibly more compact shape both for raw images (Figure S3F) and *ab initio* class averages (Figure S3G) with a maximal length for the most elongated projections of 7 nm as opposed to 12–14 nm observed with unphosphorylated MBP-ddRB-NP. Three-dimensional analysis from a dataset of 2788 molecular images was performed using an initial reference 3D map derived from the atomic model of phosphorylated ddRB-NP (PDB code 4ELJ), [21], low pass filtered to 40 Å. The resulting 3D reconstruction (Figure 3G, H) with an estimated resolution of 24 Å shows considerably enhanced detail compared to the original reference (Figure S4A iii). Segmentation of the 3D reconstruction using the Chimera segmentation procedure indicated the presence of five major domains. The MBP tag (green density in Figure 3J, K) is readily identified by comparison with the reference structure from which it is absent (Figure S4, compare iii and iv). The proposed inactive model of ddRB-NP, (PDB code 4ELJ) [21]], could be docked into the remaining density and accounted well for the remaining four segmented domains which can be recognised as the individual subdomains of RB-N and RB-P, coloured accordingly in Figure 3J and K (RB-N in blue and RB-P in pink). Comparing this docked structure with that deduced for the unphosphorylated form (Figure 3C and F) there appears to be a substantial conformational rearrangement such that active and inactive RB have a distinct architecture and relative domain arrangement.

## Discussion

We used SAXS and single particle analysis of TEM images to obtain structural models for an RB1 fragment containing the RB-N and RB-P functional regions which together make up the folded core of full length RB1. Our data reveal that in its unphosphorylated form this RB1 fragment has an elongated architecture, which upon phosphorylation of residues within the sequence connecting RB-N and RB-P converts to a compact globular conformation.

The EOM reconstruction performed using SAXS data for unmodified RB-NP (Figure 2) is consistent with the majority of molecular species adopting an elongated conformation with an Rg significantly larger than that of the compact species of ddRB-NP identified by [21] and suggests that unmodified RB-NP as analysed here adopts a preferred and stably elongated conformation. Our observations hence do not appear to support the suggestion that unmodified RB-NP exists in an equilibrium between elongated and compact conformations, and that phosphorylation shifts this equilibrium by stabilising the compact form [21].

Docking of the crystallographic structures of the RB-N and RB-P domains into TEM derived reconstructions of unmodified RB-NP permitted predictions as to the positioning of the domains and their relative orientation. Rigid body fits indicate a recessed lengthwise alignment of the domains with the various functional surfaces involved in the docking of short peptide motifs clearly accessible. In the preferred model the different functional surfaces nestle together less then 20 Å apart from each other (Figure 4). This arrangement is consistent with a model whereby interactions involving these surfaces may be cooperative or functionally coupled either to facilitate complex nucleation by bringing individual components into close proximity, or, alternatively combinatorial use of the different surfaces to select partner proteins for interaction. The available biochemical observations provide evidence that the latter mechanism has considerable relevance to the interaction of RB1 with known partner proteins. Thus multiple proteins interacting with the LXCXE binding surface in RB-P also interact with RB-N [17], [25], [31], [32], [33], [34] whilst others including the HPV E7 transforming protein simultaneously occupy the LXCXE and EXXXDLF docking surfaces [35]. Combinatorial use of interaction surfaces may increase accuracy in partner protein selection and combinatorial use of interaction surfaces could aid the assembly of distinct non-overlapping functional complexes.

An alternative model for the domain arrangement in unmodified RB-NP, featuring an inverted position of RB-N with respect to RB-P, (Figure S6) was found to match the reconstruction of the EM data reasonably well. However, this interpretation was considered to be unlikely both because residues connected by short linking sequences are separated by large distances and because the observed interactions with partner proteins described would no longer be explained by the grouping of functional sites.

Comparison of the domain arrangements in the active unmodified MBP-ddRB1-NP modelled using the crystal structures of the unmodified individual domains (PDB codes 2QDJ and 3POM) and the phosphate-modified RB-NP-domain assembly (PDB code 4ELJ) allows us to propose a mechanism by which the active conformation is converted into the inactive conformation (see Figure 4E). In the preferred fit for unphosphorylated MBP-ddRB-NP, a segment containing residues 346–355 known to be helical in unmodified RB-N (2QDJ.pdb) but disordered in inactive RB-NP (4ELJ.pdb) is positioned in the RB-N:P interface between the A cyclin folds of the respective domains (Figure 4A, C). Hence this segment is suitably located to stabilise the alignment of these domains when structured and similarly well placed to destabilize this arrangement when structurally disordered as a consequence of phosphorylation. Accordingly, an attractive proposal would be that phosphorylation of Thr356 or Thr 373 in the RBN:P joining linker which immediately follows on from the helical segment leads to its unfolding and the consequential rearrangement of RB domains into the inactive conformation (Figure 4).

Four clusters of residues ([RB-N K136, D139, T140, T142, D145], [RB-P Q736, E737, K740, K729], [RB-N L161, K164, L206-E209, L211-I213, F216, E282, E287, N290, N295] and [RB-P Q736, E737, K740, K729]) participating in the N:P interphase in inactive RB-NP [21], (Figure 4B) are predicted to be surface accessible and some distance removed from each other in our preferred model of the active conformation (Figure 4A). Furthermore, the surface involved in docking LXCXE motif interactors aligns with the evolutionarily homologous surface of RB-N in unmodified RB-NP, but these same surfaces are disjoint, facing opposing directions in inactive RB-NP [21]. Together these observations support a quite detailed model for the regulation by phosphorylation whereby domain rearrangement culminates in generating the inactive conformation of RB1.

## Materials and Methods

### RB1 Constructs

Fragments of human *RB1* cDNA (NM_000321) encoding residues 40–787 (RB-NP and MBP-RB-NP) or residues 40–787 with deletions of residues 250–269 and 579–643 (ddRB-NP and MBP-ddRB-NP) were cloned into a modified pET30 (Novagen), pET30-MBP, containing a maltose binding protein (MBP) followed by a TEV cleavage site and yielding an NH2-terminal hexahistidine–MBP tag (obtained from Laurence Pearl, Sussex). ddRB-NP was further cloned into pETM10-CMBP featuring an N-terminal hexahistidine-tag and a C-terminal MBP-tag (pETM10-CMBP). pETM10-CMBP was generated by inserting an MBP fragment produced by PCR using pET30-MBP as a template into the bacterial expression vector pETM10 (http://www.embl.de/pepcore/pepcore_services/cloning/seq/pETM-10_seq.html).

### RB1 Fragments Expression and Purification

Proteins were expressed in the Escherichia coli strain Rosetta (DE3) pLysS. Production was induced using 0.2 mM IPTG (isopropyl b-D-thiogalactopyranoside) at 20°C overnight. Bacterial cell pellets were resuspended in lysis buffer (20 mM Tris-Cl pH 7.5, 500 mM NaCl, 5 mM β-mercaptoethanol) containing EDTA-free protease inhibitor mix (Roche), lysozyme and DNase I (Roche) and suspensions sonicated. Proteins were purified from the soluble fraction by nickel-nitrilotriacetic acid affinity chromatography and eluted with lysis buffer containing 400 mM imidazole. Eluates were dialysed against buffer containing 200 mM NaCl, 20 mM Tris–HCl pH 7.5, 10 mM β-mercaptoethanol and 1 mM EDTA. Proteins were further purified by amylose affinity chromatography using a 5 ml MBP-Trap column (GE Healthcare), with elution into dialysis buffer containing 20 mM maltose, followed by size-exclusion chromatography using a Superdex 200 16/60 column (GE Healthcare) pre-equilibrated with 20 mM Tris-Cl pH 7.5, 200 mM NaCl and 10 mM β-mercaptoethanol. MBP-tags were removed using TEV protease prior to size exclusion chromatography where indicated. Samples were concentrated by using a VivaSpin20 concentrator MWCO 30.000 (Sartorius). The protein purity was examined by SDS–PAGE electrophoresis. The same expression and purification procedure was used for all constructs.

### Enzymatic Modifications

Purified RB1 protein preparations were phosphorylated in a reaction containing 10 mM MgCl2, 10 mM ATP, 100 mM NaCl, 25 mM Tris-CL (pH 8.0), and 2% (volume per mass) Kaposi’s Sarcoma-associated herpesvirus cyclin (K cyclin) activated Cdk6. K cyclin-activated Cdk6 were produced by recombinant baculovirus infection of SF9 insect cells as described [36]. Reaction conditions were essentially as in [37], except that reactions were performed at 4°C, for a total of 60 min.

Kinase-treated MBP-ddRB-NP was purified using amylase affinity chromatography followed by size exclusion chromatography. To confirm phosphorylation proteins were subjected to LC/MS/MS analysis using a LTQ Velos Orbitrap mass spectrometer (Thermo Fisher Scientific, Hemel Hempstead, UK) fitted with a non-coated SilicaTip emitter (20 µm I.D., 10 µm tapered tip; New Objectives, Woburn, MA, USA). MS/MS-based spectra were mined using Scaffold v3.0 (Proteome Software Inc., Portland, OR).

### SAXS Data Collection and Analysis

Synchrotron SAXS data were collected on the EMBL X33 camera on the storage ring DORIS III (Deutsches Elektronen-Synchrotron (DESY), Hamburg, Germany) using either a MAR345 IP detector and 4 frames with exposure time of 30 seconds or a Pilatus 1 M pixel detector and eight frames with exposure time of 15-seconds. The sample-to-detector distance was 2.7 m, covering a range of momentum transfer 0.1 nm^−1^<*s*<6 nm^−1^ (*s*  = 4πsinθ/λ, where 2 θ is the scattering angle, and λ  = 0.15 nm is the X-ray wavelength). Comparison of successive 15-second or 30-second frames revealed no evidence for radiation damage. All samples were measured at a minimum of two solute concentrations ranging from 1 mg/ml to 4 mg/ml at 4°C. The subtracted curves were scaled against the solute concentrations and either merged (in the absence of concentration dependence) or extrapolated to infinite dilution (in the presence of concentration dependence) using *PRIMUS* [22]. The radius of gyration R_g_ and forward scattering I(0), the maximum particle dimension D_max_ and the distance distribution function p(r) were evaluated using the program GNOM [38]. Molecular masses of solutes were estimated by calibration against reference solutions of bovine serum albumin. The excluded particle volume Vp was computed from the scattering data using Porod invariant [39]. Low resolution *ab initio* models were generated using DAMMIN [19]. The results of 10 independent DAMMIN runs were analyzed and averaged by SUPCOMB [40] and DAMAVER [41].

The position of MBP in the fusion protein was determined by *ab initio* program MONSA [19], an extended version of DAMMIN. In this approach, scattering patterns from ddRB-NP MBP-ddRB-NP and ddRB-NP-MBP were simultaneously fitted by the multiphase bead model depicting the ddRB-NP and MBP moieties in these constructs.

### EM Methods

Both MBP-ddRB-NP and phosphorylated MBP-ddRB-NP samples were applied at a concentration of ∼10 µg/ml to glow-discharged quantifoil grids coated with a continuous thin carbon layer and stained with 2% uranyl acetate. Images were collected with a FEI Tecnai F20 electron microscope operating at 200 kV on a Tietz 4 k×4 k CCD detector at a nominal magnification of x 50,000 corresponding to a sampling rate of 3.47 Å/pixel. Single molecular views were chosen manually using Boxer, part of EMAN [42], resulting in an initial data set of 5829 particles for MBP-ddRB-NP and 2788 for phosphorylated MBP-ddRB-NP. Reference-free class averages were obtained on the datasets band pass filtered between 150 and 30 Å using the procedure refine2d from EMAN and compared with the refined class averages obtained during the 3D analysis. Subsequent processing used programs from Imagic [43] and SPIDER [44].

For the 3D analysis the datasets were band pass filtered between 150 and 20 Å. Initial reference models were generated from the SAXS envelope of MBP-ddRB-NP and the crystal structure of phosphorylated MBP-ddRB-NP (PDB code 4ELJ); [21]. Both models were low-pass filtered to 40 Å and used to align the respective experimental datasets. Angular assignment was performed by projection matching in Imagic and the 3D reconstructions were calculated using a locally developed Fourier space algorithm [45]. The refinement consisted of iterations of multi-reference alignment in SPIDER, multivariate statistical analysis and classification in Imagic. At each stage the agreement between class and reprojection was assessed and a manual selection of classes composed of a homogeneous population of single molecular images was made in order to calculate a new 3D volume and its forward projections used as references for the next cycle of refinement. The final 3D map of MBP-ddRB-NP was obtained from 2941 particles and that of phosphorylated MBP-ddRB-NP from 2523. The maps were segmented using an automatic procedure in Chimera [46].

## Supporting Information

Figure S1 **Characterisation of RB1-derivative preparations. A.** Multi-angel light scattering (MALS) molar mass distribution plot. Data were recorded in flow mode. RB preparations are colour-coded as indicated. Horizontal lines represented the molecular weight obtained as a function of the elution volumes. **B.** ddRB-NP was cross-linked with BS3 and analyzed by SDS-Polyacrylamide gel elecrtophoresis. Marker (lane 1), ddRB-NP (lane 2), ddRB-NP samples cross-linked with 25 mM, 5 mM, 0.5 mM and 0.05 mM Bis[sulfosuccinimidyl] suberate (BS3), respectively **(**lanes 3–4), at a protein concentration of 3 mg/ml. **C.** Guinier region plot for samples as indicated, at C1 concentration. For derived parameters refer to Table S2.
(TIFF)

Figure S2 **SAXS results for ddRB-NP derivatives and corresponding RB-NP derivatives.** **A.** Experimental scattering patterns of (1) ddRB-NP shown as red triangles with black error bars and RB-NP, shown as black squares with grey error bars, and (2) MBP-ddRB-NP shown as red triangles with black error bars and MBP-RB-NP shown as black squares with grey error bars. Shown is the logarithm of the scattering intensity as a function of momentum transfer s = 4πsin(θ/2)/λ where θ is the scattering angle and λ  = 1.5 Å is the X-ray wavelength. **B.** Distance distribution functions for constructs ddRB-NP, RB-NP, MBP-ddRB-NP and MBP-RB-NP.
(TIFF)

Figure S3 **Electron microscopy of MBP-ddRB-NP. A.–E.** unmodified MBP-ddRB-NP **A.** Electron micrograph of a negatively stained MBP-ddRB-NP. Different views are identified with black circles. **B.** Selection from the initial class averages obtained by automated alignment and classification procedures. **C.** Examples of single particles (i), their corresponding class average (ii) and re- projections of the 3D reconstruction in their assigned orientation (iii). **D.** Distribution of Euler angles. **E.** Resolution assessment by Fourier shell correlation showing a resolution of 27 Å at 0.5 correlation. **F.–J. phosphorylated MBP-ddRB-NP F.** Electron micrograph of a negatively stained phosphorylated MBP-ddRB-NP. Different views are identified with black circles. **G.** Selection from the initial class averages obtained by automated alignment and classification procedures. **H.** Examples of single particles (i), their corresponding class average (ii) and re- projections of the 3D reconstruction in their assigned orientation (iii). **I.** Distribution of Euler angles. **J.** Resolution assessment by Fourier shell correlation showing a resolution of 24 Å at 0.5 correlation.
(TIFF)

Figure S4 **Surface views of the initial and final 3D models of MBP-ddRB-NP and phosphorylated MBP-ddRB-NP. A**. 3D reconstruction for unmodified MBP-ddRB-NP (i, ii) and phophorylated MBP-ddRB-NP (ii, iv) (i) Surface view of the 3D volume derived by converting a SAXS envelope of MBP-ddRB-NP followed by low pass filtering to 40 Å. (ii) Surface view of the 3D reconstruction of MBP-ddRB-NP obtained using the forward projections of the model shown in (i) as reference for initial alignment and projection matching. The obtained 3D map is consistent with the model but exhibits more features than the starting model (i). (iii) Surface view of the 3D volume derived by low pass filtering the atomic model 4ELJ.pdb to 40 Å. (iv) Surface view of the 3D reconstruction of phosphorylated MBP-ddRB-NP obtained using the forward projections of the model shown in (iii) as reference for initial alignment and projection matching. The 3D reconstruction is more detailed compared with (iii) and density for the MBP-tag (absent from the initial model) is visible. **B.** Three orthogonal surface views of unmodified MBP-ddRB-NP **C.** Three orthogonal surface views of phosphorylated MBP-ddRB-NP. Maps in B) and C) were aligned manually in Chimera with respect to their respective RB-N densities.
(TIFF)

Figure S5 **PHYRE-based** ***in silico*** **model prediction for RB-NP. A.** PHYRE2 generated model for RB-NP, R-Linker (residues 250–269) and P-linker (residues 579–643) are depicted in black, the sequence joining RB-N and RB-P (residues 355–357) is coloured in red, RB-N lobe A in cyan, lobe B in blue. RB-P lobe A in dark salmon, RB-P lobe B in pink. The most likely model obtained is shown, with residues (67%) modelled at >90% accuracy. Modelling was performed prior to knowledge of 4ELJ. **B.** Proposed domain orientation for unmodified RB1, based on single particle EM, from Figure 3, displayed for comparison.
(TIFF)

Figure S6 **Model alternative for domain arrangement. A.** Alternatively docked structures of RB-N and RB-P (2QDJ and 3POM) (i) superimposed on the 3D single particle reconstruction from TEM images. The calculated density map is shown in mesh representation in grey, RB-N lobe A in cyan, lobe B in light blue. RB-P lobe A is shown in dark salmon, RB-P lobe B in pink, as for Figure 3. The alternatively docked model requires assumptions that linkers joining RB-N and RB-P (residue 356–374) and linking RB-N to MBP adopt a maximally extended, unstructured conformation (>2.5A/peptide bond). Surface distance estimations between adjoining residues in individual domains are indicated, residue positions are marked with blue-filled circles, * denotes unoccupied density. The initial favoured model from Figure 3 with distance estimation shown for comparison (ii). **B.** Positioning of functional surfaces in alternative (i) and preferred (ii) model for active RB-NP. Surface model superimposed with cartoon. RB-N in light blue, RB-P in light-pink, residues involved in docking LXCXE in yellow, FXXXV in purple, EXXXDLFD in cyan, residues 346–355 which are structured in unmodified RB-N but unstructured in inactive RB-NP in grey, amino acid groups involved in the RB-N:P interphase in the inactive conformation in red ([RB-N K136, D139, T140, T142, D145], [RB-P Q736, E737, K740, K729]) and orange [(RB-N L161, K164, L206- E209, L211-I213, F216, E282, E287, N290, N295] [RB-P Q736, E737, K740, K729]). **C.** Simulation of molecular movement required to generate the inactive conformation based on the alternative model, necessitating rotation around a centrally located axis within RB-N along with a 20 Å descend to align domains as in the inactive conformation.
(TIF)

Figure S7 **MS/MS characterisation of Kcyclin/cdk6 phosphorylated MBP-ddRB-NP. A.** Documentation of sequence coverage. Yellow regions indicate peptide coverage. Proline-directed consensus sites within RB1 (Ser230, Thr356, Thr373 an Ser780) are boxed **B., C.** MS/MS collision spectra identifying phosphorylation on RB1 residue T356 (B) and T373 (C). Recorded y and b ions and the related peptide sequence are labelled, graphs depicting mass/charge versus intensity.
(TIFF)

Table S1 **Theoretical and experimentally determined molecular weights (MW) for proteins in solution based on multi-angle light-scattering (MALS).**
(DOC)

Table S2 **SAXS-derived parameters for datasets used in this study.**
(DOC)
