# Supplementary figures and images for: Structural Insights into the Mechanism of Phosphoregulation of the Retinoblastoma Protein
Source: PLoS One. 2013 Mar 14;8(3):e58463. doi: 10.1371/journal.pone.0058463 (PMC3597711; doi:10.1371/journal.pone.0058463)

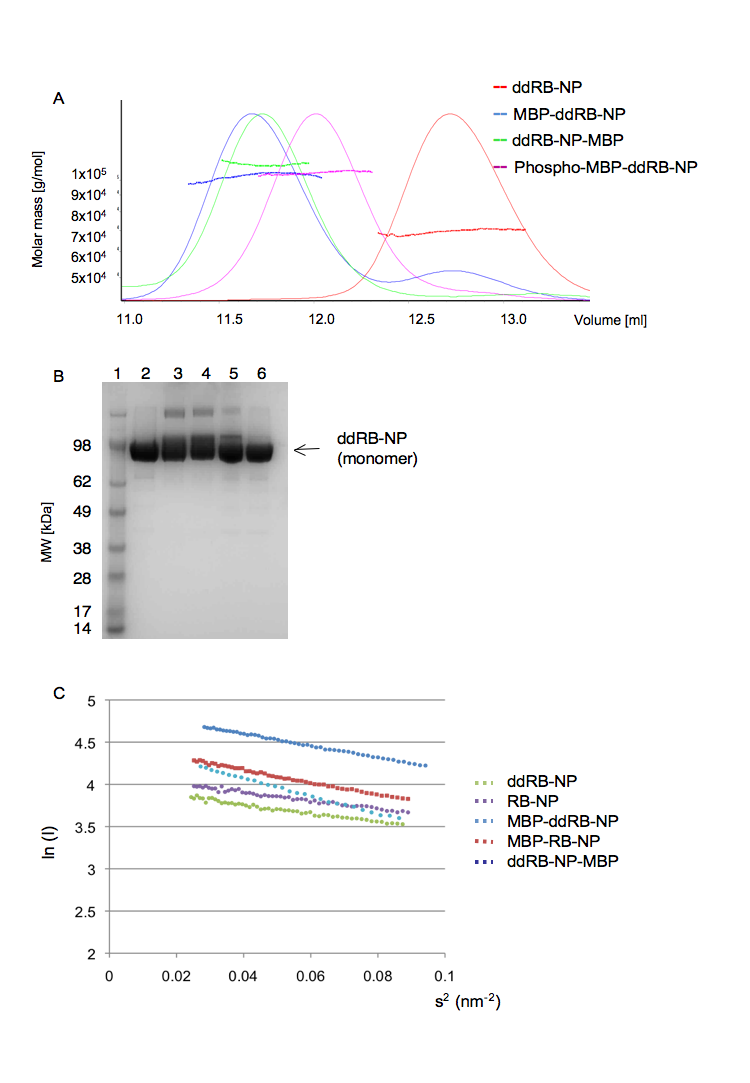

Supplement: Figure S1 — Characterisation of RB1-derivative preparations. A. Multi-angel light scattering (MALS) molar mass distribution plot. Data were recorded in flow mode. RB preparations are colour-coded as indicated. Horizontal lines represented the molecular weight obtained as a function of the elution volumes. B. ddRB-NP was cross-linked with BS3 and analyzed by SDS-Polyacrylamide gel elecrtophoresis. Marker (lane 1), ddRB-NP (lane 2), ddRB-NP samples cross-linked with 25 mM, 5 mM, 0.5 mM and 0.05 mM Bis[sulfosuccinimidyl] suberate (BS3), respectively (lanes 3–4), at a protein concentration of 3 mg/ml. C. Guinier region plot for samples as indicated, at C1 concentration. For derived parameters refer to Table S2. (TIFF) [file pone.0058463.s001.tif]

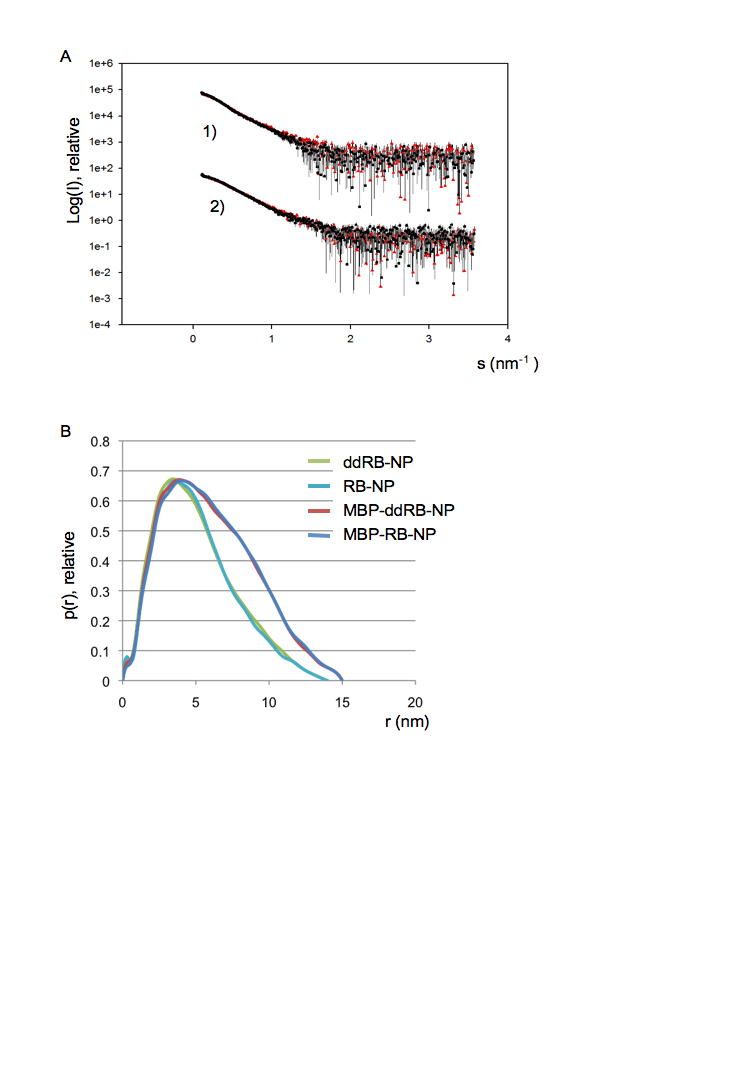

Supplement: Figure S2 — SAXS results for ddRB-NP derivatives and corresponding RB-NP derivatives. A. Experimental scattering patterns of (1) ddRB-NP shown as red triangles with black error bars and RB-NP, shown as black squares with grey error bars, and (2) MBP-ddRB-NP shown as red triangles with black error bars and MBP-RB-NP shown as black squares with grey error bars. Shown is the logarithm of the scattering intensity as a function of momentum transfer s = 4πsin(θ/2)/λ where θ is the scattering angle and λ = 1.5 Å is the X-ray wavelength. B. Distance distribution functions for constructs ddRB-NP, RB-NP, MBP-ddRB-NP and MBP-RB-NP. (TIFF) [file pone.0058463.s002.tif]

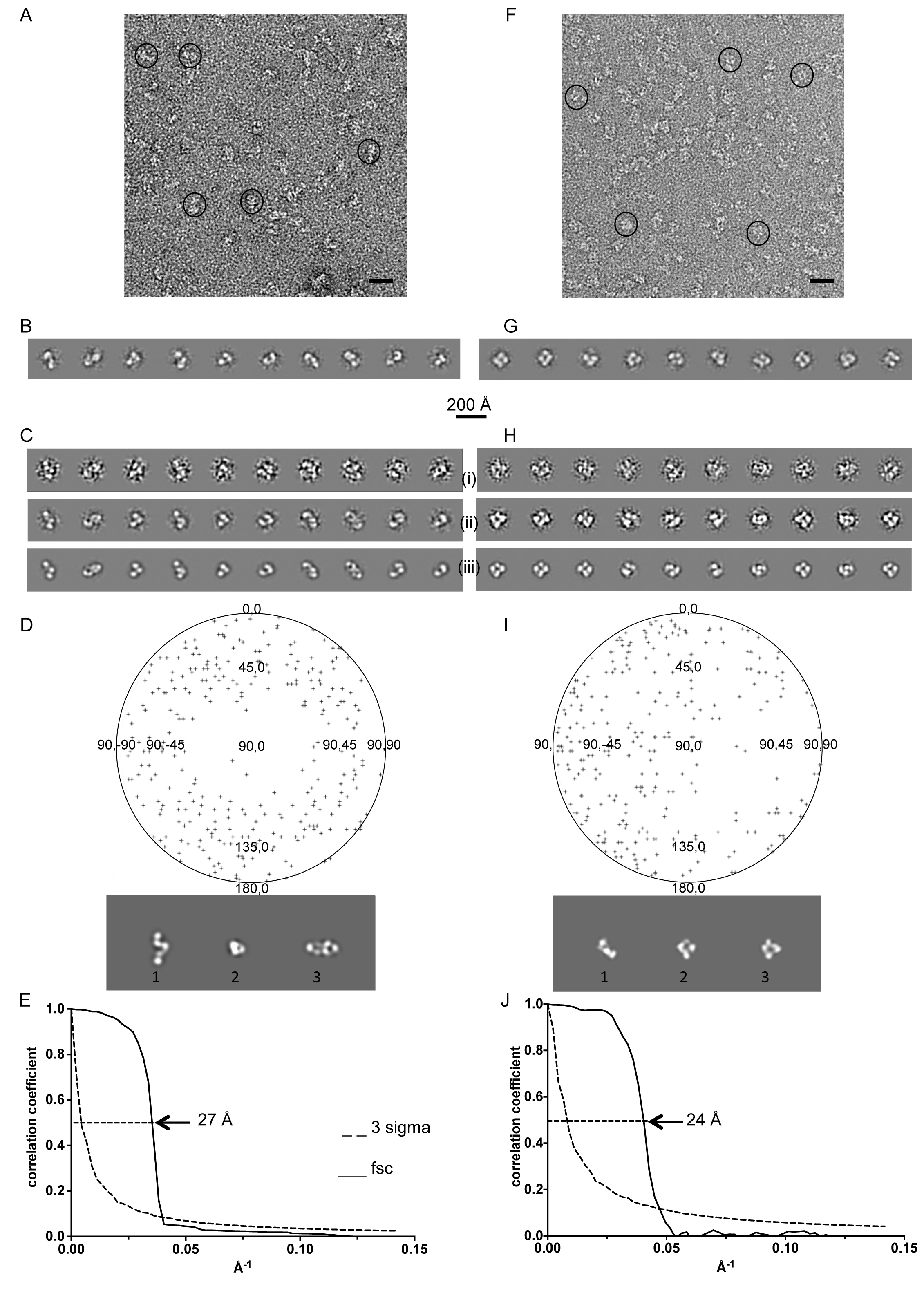

Supplement: Figure S3 — Electron microscopy of MBP-ddRB-NP. A.–E. unmodified MBP-ddRB-NP A. Electron micrograph of a negatively stained MBP-ddRB-NP. Different views are identified with black circles. B. Selection from the initial class averages obtained by automated alignment and classification procedures. C. Examples of single particles (i), their corresponding class average (ii) and re- projections of the 3D reconstruction in their assigned orientation (iii). D. Distribution of Euler angles. E. Resolution assessment by Fourier shell correlation showing a resolution of 27 Å at 0.5 correlation. F.–J. phosphorylated MBP-ddRB-NP F. Electron micrograph of a negatively stained phosphorylated MBP-ddRB-NP. Different views are identified with black circles. G. Selection from the initial class averages obtained by automated alignment and classification procedures. H. Examples of single particles (i), their corresponding class average (ii) and re- projections of the 3D reconstruction in their assigned orientation (iii). I. Distribution of Euler angles. J. Resolution assessment by Fourier shell correlation showing a resolution of 24 Å at 0.5 correlation. (TIFF) [file pone.0058463.s003.tif]

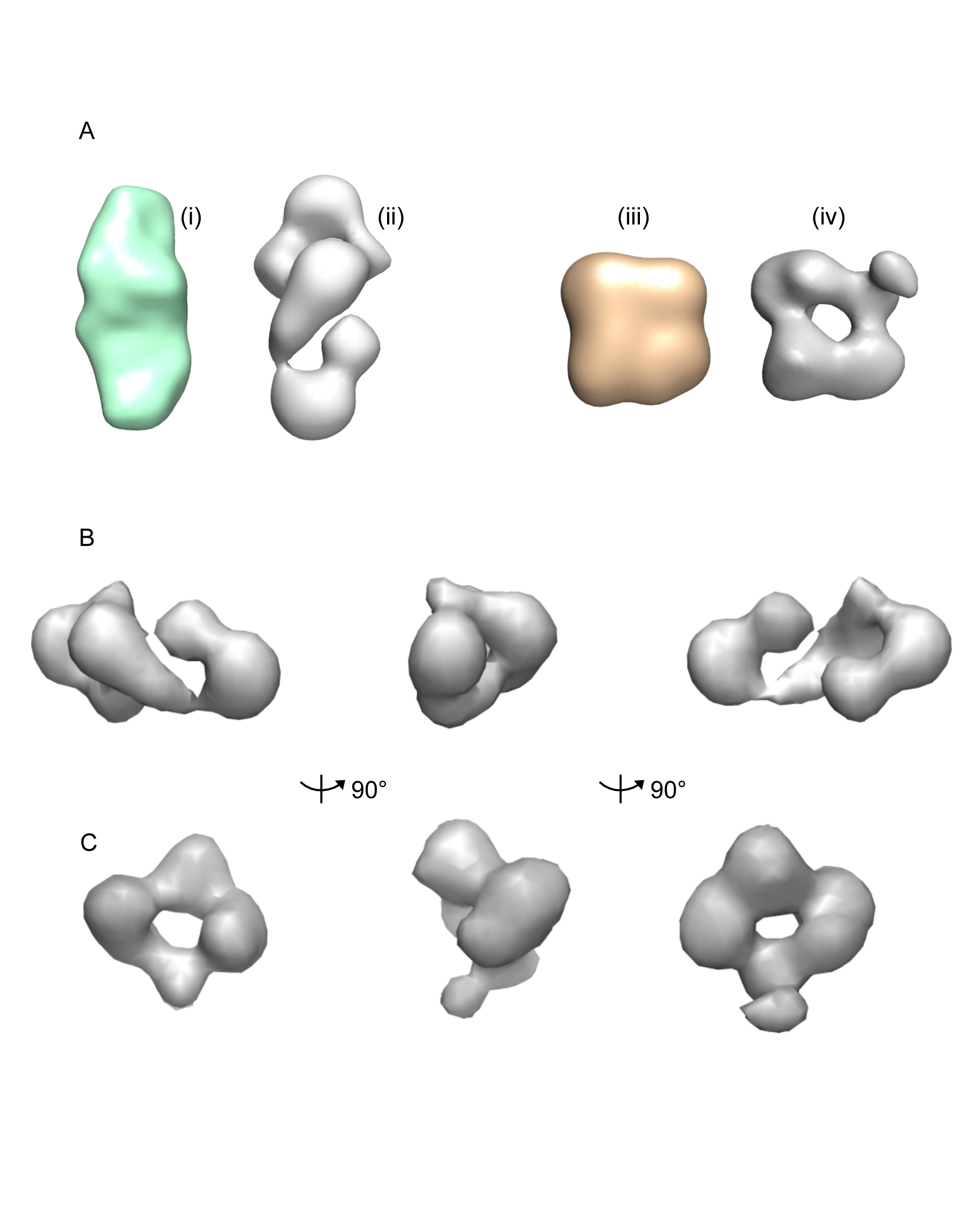

Supplement: Figure S4 — Surface views of the initial and final 3D models of MBP-ddRB-NP and phosphorylated MBP-ddRB-NP. A. 3D reconstruction for unmodified MBP-ddRB-NP (i, ii) and phophorylated MBP-ddRB-NP (ii, iv) (i) Surface view of the 3D volume derived by converting a SAXS envelope of MBP-ddRB-NP followed by low pass filtering to 40 Å. (ii) Surface view of the 3D reconstruction of MBP-ddRB-NP obtained using the forward projections of the model shown in (i) as reference for initial alignment and projection matching. The obtained 3D map is consistent with the model but exhibits more features than the starting model (i). (iii) Surface view of the 3D volume derived by low pass filtering the atomic model 4ELJ.pdb to 40 Å. (iv) Surface view of the 3D reconstruction of phosphorylated MBP-ddRB-NP obtained using the forward projections of the model shown in (iii) as reference for initial alignment and projection matching. The 3D reconstruction is more detailed compared with (iii) and density for the MBP-tag (absent from the initial model) is visible. B. Three orthogonal surface views of unmodified MBP-ddRB-NP C. Three orthogonal surface views of phosphorylated MBP-ddRB-NP. Maps in B) and C) were aligned manually in Chimera with respect to their respective RB-N densities. (TIFF) [file pone.0058463.s004.tif]

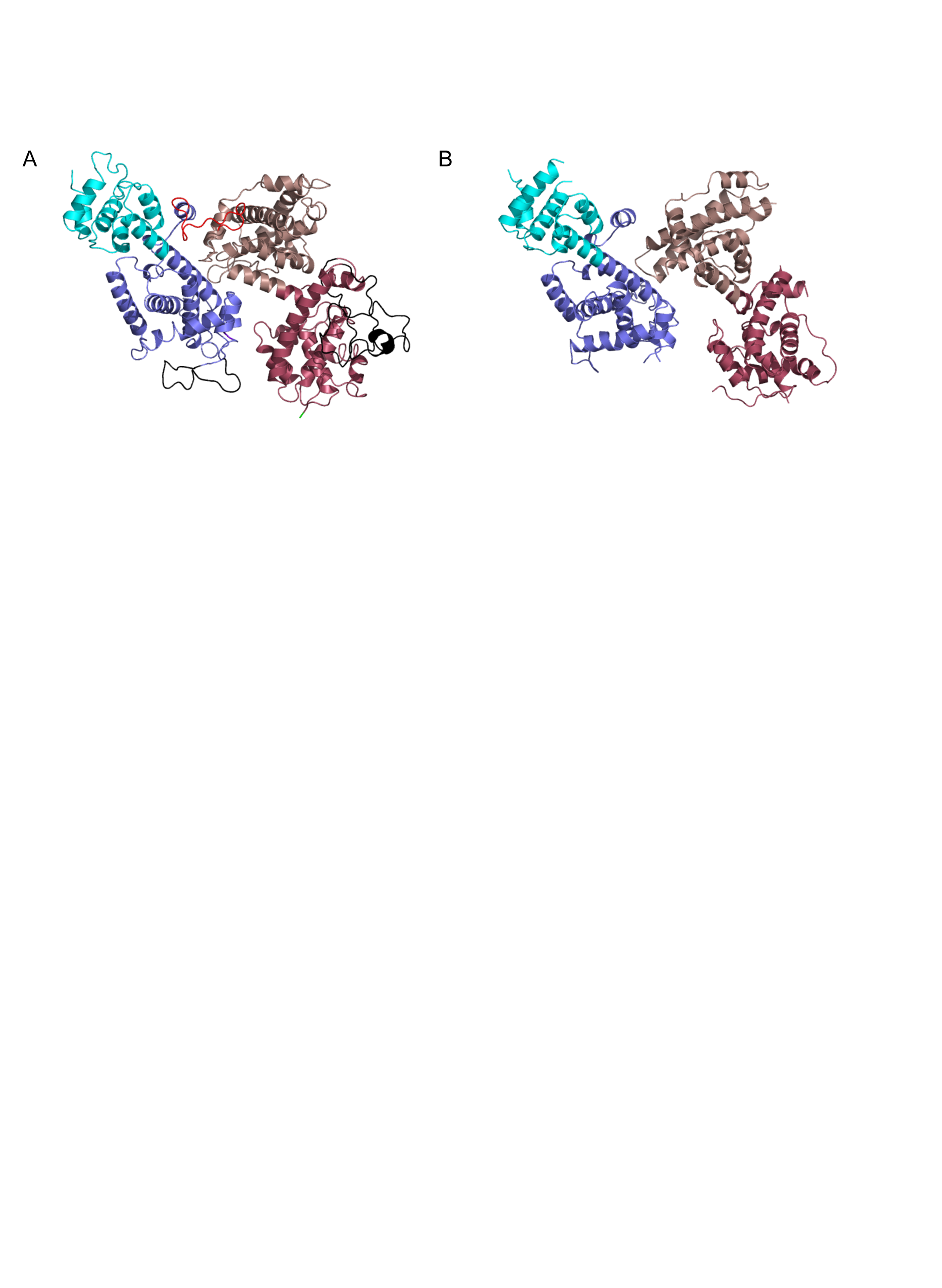

Supplement: Figure S5 — PHYRE-based in silico model prediction for RB-NP. A. PHYRE2 generated model for RB-NP, R-Linker (residues 250–269) and P-linker (residues 579–643) are depicted in black, the sequence joining RB-N and RB-P (residues 355–357) is coloured in red, RB-N lobe A in cyan, lobe B in blue. RB-P lobe A in dark salmon, RB-P lobe B in pink. The most likely model obtained is shown, with residues (67%) modelled at >90% accuracy. Modelling was performed prior to knowledge of 4ELJ. B. Proposed domain orientation for unmodified RB1, based on single particle EM, from Figure 3, displayed for comparison. (TIFF) [file pone.0058463.s005.tif]

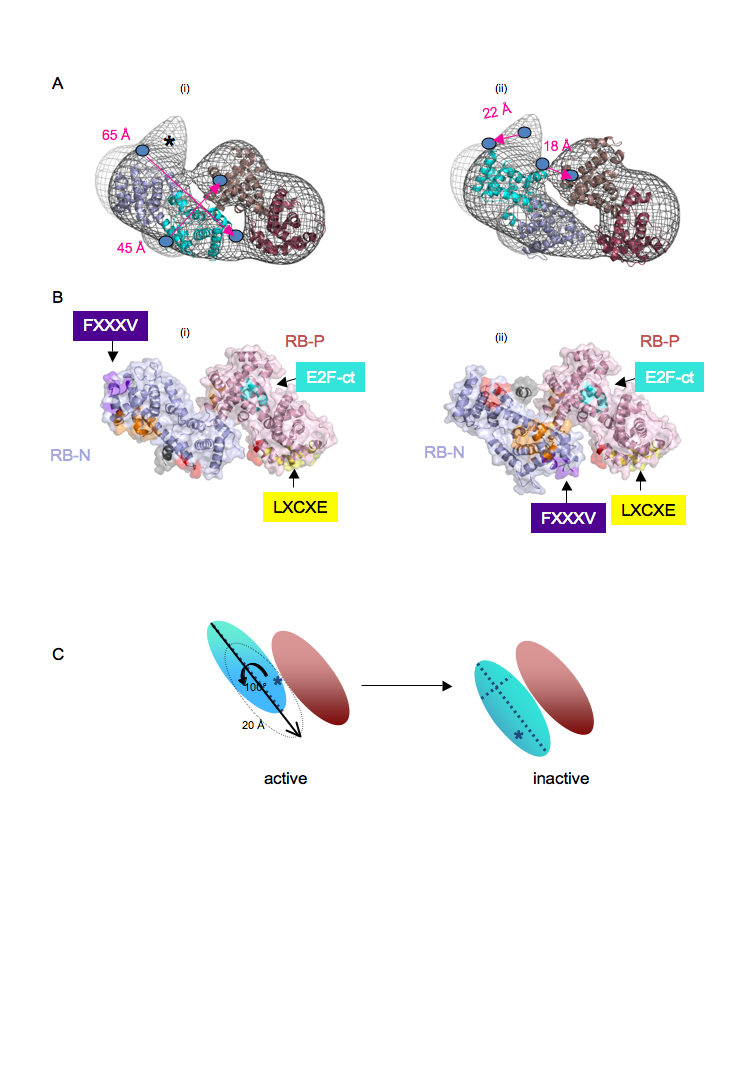

Supplement: Figure S6 — Model alternative for domain arrangement. A. Alternatively docked structures of RB-N and RB-P (2QDJ and 3POM) (i) superimposed on the 3D single particle reconstruction from TEM images. The calculated density map is shown in mesh representation in grey, RB-N lobe A in cyan, lobe B in light blue. RB-P lobe A is shown in dark salmon, RB-P lobe B in pink, as for Figure 3. The alternatively docked model requires assumptions that linkers joining RB-N and RB-P (residue 356–374) and linking RB-N to MBP adopt a maximally extended, unstructured conformation (>2.5A/peptide bond). Surface distance estimations between adjoining residues in individual domains are indicated, residue positions are marked with blue-filled circles, * denotes unoccupied density. The initial favoured model from Figure 3 with distance estimation shown for comparison (ii). B. Positioning of functional surfaces in alternative (i) and preferred (ii) model for active RB-NP. Surface model superimposed with cartoon. RB-N in light blue, RB-P in light-pink, residues involved in docking LXCXE in yellow, FXXXV in purple, EXXXDLFD in cyan, residues 346–355 which are structured in unmodified RB-N but unstructured in inactive RB-NP in grey, amino acid groups involved in the RB-N:P interphase in the inactive conformation in red ([RB-N K136, D139, T140, T142, D145], [RB-P Q736, E737, K740, K729]) and orange [(RB-N L161, K164, L206- E209, L211-I213, F216, E282, E287, N290, N295] [RB-P Q736, E737, K740, K729]). C. Simulation of molecular movement required to generate the inactive conformation based on the alternative model, necessitating rotation around a centrally located axis within RB-N along with a 20 Å descend to align domains as in the inactive conformation. (TIF) [file pone.0058463.s006.tif]

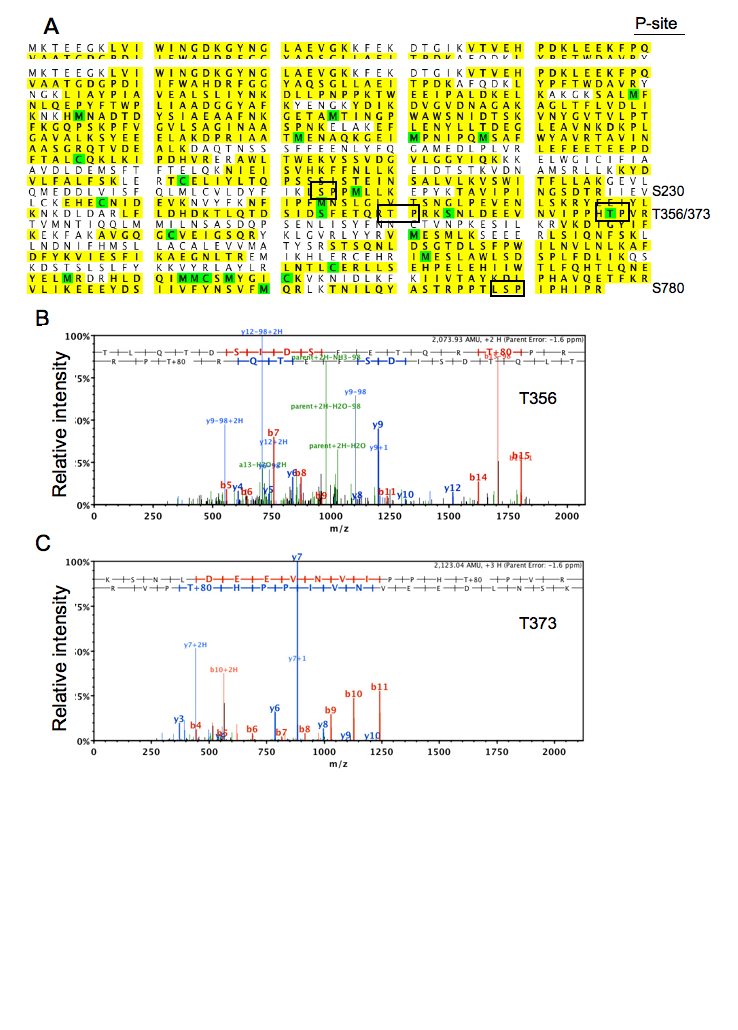

Supplement: Figure S7 — MS/MS characterisation of Kcyclin/cdk6 phosphorylated MBP-ddRB-NP. A. Documentation of sequence coverage. Yellow regions indicate peptide coverage. Proline-directed consensus sites within RB1 (Ser230, Thr356, Thr373 an Ser780) are boxed B., C. MS/MS collision spectra identifying phosphorylation on RB1 residue T356 (B) and T373 (C). Recorded y and b ions and the related peptide sequence are labelled, graphs depicting mass/charge versus intensity. (TIFF) [file pone.0058463.s007.tif]
