# Supplementary material for: Structural Insights into the Mechanism of Phosphoregulation of the Retinoblastoma Protein
Source: PLoS One. 2013 Mar 14;8(3):e58463. doi: 10.1371/journal.pone.0058463 (PMC3597711; doi:10.1371/journal.pone.0058463)
Supplement: Table S1 — Theoretical and experimentally determined molecular weights (MW) for proteins in solution based on multi-angle light-scattering (MALS). (DOC) [file pone.0058463.s008.doc]

| **Sample** | **Theoretical MW (kDa)** | **Experimental MW (kDa)**  **obtained by MALS** |
| --- | --- | --- |
| ddRB-NP | 77.14 | 70.8 ± 2.8 |
| MBP-ddRB-NP | 115.1 | 105.0 ± 2.5 |
| ddRB-NP-MBP | 112.5 | 107.0 ± 2.5 |
| Phospho MBP-ddRB-NP | 115.1 | 105.8 ± 0.8 |

Table S1**.** Theoretical and experimentally determined molecular weights (MW) for proteins in solution based on multi-angle light-scattering (MALS).
