# Supplementary material for: Structural Insights into the Mechanism of Phosphoregulation of the Retinoblastoma Protein
Source: PLoS One. 2013 Mar 14;8(3):e58463. doi: 10.1371/journal.pone.0058463 (PMC3597711; doi:10.1371/journal.pone.0058463)
Supplement: Table S2 — SAXS-derived parameters for datasets used in this study. (DOC) [file pone.0058463.s009.doc]

| **Sample** | **Sample concentration** | **Rg (nm)** | **Dmax (nm)** | **Excluded**  **volume** **(nm^3^)** |
| --- | --- | --- | --- | --- |
| ddRB-NP | 1.4 mg/ml | 4.1 ± 0.1 | 14.0 ± 1 | 142 ± 2 |
| ddRB-NP | 2.7 mg/ml | 4.0 ± 0.1 | 14.0 ± 1 | 149 ± 2 |
| RB-NP | 1.2 mg/ml | 3.8 ± 0.1 | 14.0 ± 1 | 179 ± 2 |
| RB-NP | 2.4 mg/ml | 4.0 ± 0.1 | 14.0 ± 1 | 179 ± 2 |
| MBP-ddRB-NP | 1.1 mg/ml | 4.5 ± 0.1 | 15.0 ± 1 | 175 ± 2 |
| MBP-ddRB-NP | 2.2 mg/ml | 4.6 ± 0.1 | 15.0 ± 1 | 233 ± 2 |
| MBP-RB-NP | 1.7 mg/ml | 4.6 ± 1 | 15.0 ± 1 | 238 ± 2 |
| MBP-RB-NP | 2.8 mg/ml | 4.6 ± 0.1 | 15.0 ± 1 | 227 ± 2 |
| ddRB-NP-MBP | 1.6 mg/ml | 5.2 ± 0.1 | 17 ± 1 | 152 ± 2 |
| ddRB-NP-MBP | 4.2 mg/ml | 5.1 ± 0.1 | 18 ± 1 | 154 ± 2 |

**Table S2.** SAXS-derived parameters for datasets used in this study.
